# Higher pain catastrophizing scale is associated with more postoperative pain within the first week after rotator cuff repair

**DOI:** 10.1002/jeo2.70359

**Published:** 2025-07-13

**Authors:** Alexandra M. Stein, Alexandre Hardy, Mohamad Moussa, Thomas Bauer, Jean‐David Werthel

**Affiliations:** ^1^ Service de chirurgie orthopédique Hôpital Ambroise Paré Boulogne‐Billancourt France; ^2^ Chirurgie du sport Clinique du sport Paris France

**Keywords:** analgesia, catastrophizing, pain, postoperative, rotator cuff

## Abstract

**Purpose:**

Many psychological factors play a role in the patient's reported pain after rotator cuff surgery. The aim of this study was to investigate the influence of preoperative pain catastrophizing, assessed using the Pain Catastrophizing Scale (PCS), on early postoperative pain following rotator cuff repair.

**Methods:**

A prospective case series was conducted. Patients who underwent a rotator cuff repair in our hospital were included consecutively for 6 months in 2024. The PCS was used preoperatively to evaluate patient's apprehensions about pain. The primary outcome measure was the visual analog scale (VAS) as compared between both group on postoperative Days 0–5.

**Results:**

A total of 33 patients were included in the study, with 16 patients in the low PCS group (mean PCS score of 4.6) and 16 patients in the high PCS group (mean PCS score of 25.0). Pain scores, measured using the Visual Analog Scale (VAS), were significantly higher in the high PCS group compared to the low PCS group at all time points. On Day 2 (D2), the VAS pain score was 2.0 (1.8; 4.0) in the low PCS group and 5.0 (2.0; 8.0) in the high PCS group (*p* = 0.02). On Day 3 (D3), scores were 2.0 (1.0; 3.0) and 5.5 (1.8; 7.3), respectively (*p* = 0.03). On Day 4 (D4), the scores were 2.0 (0.8; 3.0) in the low PCS group and 4.5 (1.0; 6.0) in the high PCS group (*p* = 0.03). Finally, on Day 5 (D5), pain scores were 1.5 (0.0; 2.3) for the low PCS group and 4.0 (1.0; 5.3) for the high PCS group (*p* = 0.04).

**Conclusion:**

Patients presenting high levels of catastrophizing experienced more early postoperative pain following arthroscopic rotator cuff repair surgery than patients with low levels of catastrophizing.

**Level of Evidence:**

IV.

**Abbreviations**
ASES: American Shoulder and Elbow Surgeons
BID: ‘bis in die’ (latin) = twice a day
D: Day
IQR: interquartile range
LOT‐R: Life Orientation Test‐Revised
PCS: pain catastrophizing scale
QID: ‘quarter in die’ (latin) = four times a day
SST: Simple Shoulder Test
VAS: Visual Analog Scale
WORC: Western Ontario Rotator Cuff Index

## BACKGROUND

Rotator cuff tears are a common cause of shoulder pain, with both acute and chronic presentations often linked to an underlying degenerative process. Even in cases of acute traumatic tears, pre‐existing tendon degeneration is frequently observed, highlighting the progressive nature of rotator cuff disease [16]. The majority of patients undergoing surgical repair experience chronic shoulder pain, which significantly impacts their daily activities and quality of life [1, 3, 6]. However, despite the benefits of surgery, rehabilitation following rotator cuff repair is challenging due to substantial early postoperative pain [18, 22]. While early postoperative pain is often intense (within the first 5 days), long‐term outcomes remain favourable, with 85%–90% of patients reporting no or minimal shoulder pain after completing rehabilitation, typically within 1 to 2 years postsurgery [7, 10, 12, 14, 25, 30]. It is essential to distinguish between early postoperative pain, which is an acute response to surgical trauma and inflammation, and persistent shoulder pain after full rehabilitation, which may be driven by chronic pain mechanisms such as central sensitization. Understanding these differences is crucial for optimizing postoperative care and improving patient outcomes [21, 26].

The Pain Catastrophizing scale (PCS), developed by Sullivan in 1995 is a widely used psychometric tool designed to assess an individual's cognitive and emotional response to pain. The PCS consists of 13 items that evaluate three distinct domains: rumination (persistent focus on pain‐related thoughts), magnification (exaggeration of the perceived threat of pain), and helplessness (a sense of inability to manage pain) [17]. Each item is rated on a 5‐point scale, ranging from 0 (‘Not at all’) to 4 (‘All the time’), with higher scores indicating greater levels of catastrophizing. Higher PCS scores have been consistently associated with increased pain sensitivity, greater emotional distress, and a heightened risk of developing chronic pain conditions [2].

Studies have shown that pain catastrophizing can modulate pain perception by enhancing central sensitization, altering descending pain inhibition pathways, and increasing emotional distress, all of which contribute to the transition from acute postoperative pain to chronic pain [9]. Furthermore, higher preoperative pain catastrophizing scores have been associated with greater opioid consumption, lower rehabilitation adherence, and decreased overall patient satisfaction following surgery. Given that the vast majority of patients achieve favourable long‐term outcomes after rotator cuff repair, understanding the role of psychological factors such as pain catastrophizing is critical for identifying patients at risk for prolonged pain and implementing targeted interventions to improve recovery trajectories [6, 11, 21, 26, 31, 32]. The potential impact of pain catastrophizing on early postoperative pain in rotator cuff repair remains poorly understood. Understanding how pain catastrophizing influences postoperative pain in this patient population could provide valuable insights into improving clinical outcomes.

The aim of this study was to analyze the effect of preoperative pain catastrophizing, as assessed by the PCS, on early postoperative pain as measured by the Visual Analog Scale (VAS) in patients with rotator cuff tears [24]. Our hypothesis was that patients with higher PCS scores would be more likely to experience greater postoperative pain during the early recovery period.

## METHODS

### Population

This prospective case series included 33 patients who underwent arthroscopic rotator cuff repair at our healthcare facility between November 2023 and May 2024. Inclusion criteria were: adult patients (aged 18 years or older) presenting with a full‐thickness, nonretracted posterosuperior rotator cuff tear, treated arthroscopically during the study period. In cases of chronic rotator cuff tears, surgery was considered only after failure of conservative management, which consisted of at least 6 months of structured physical therapy and at least one corticosteroid injection. Patients were eligible for surgical intervention if they remained symptomatic, with persistent weakness, limitation in daily activities, or a decline in quality of life despite nonoperative treatment.

Exclusion criteria included patients undergoing revision rotator cuff repair, those with irreparable rotator cuff tears, and those treated by open surgical approaches.

### Surgical technique

All patients underwent primary rotator cuff repair by a single attending (JDW). Anesthesia was obtained by interscalene block and general anesthesia. Patients were placed on lateral decubitus and a 3 kg weight was used to track their arm. Rotator cuff lesions were repaired using a double‐row technique. Fixation was obtained with a nonesorbable screw: Corck‐screw FT™ (Arthrex, Inc.) 5.5 mm for subscapularis repair, Biocorck‐screw FT™ (Arthrex, Inc.) 5.5 mm for supraspinatus or infraspinatus repair, Swive‐lock™ anchor (Arthrex, Inc.) anchor to apply the lateral row. Biceps tenotomy or tenodesis was based on the patients' age and functional demands. Tenodesis was preferred for young, lean and athletic patients. An acromioplasty was performed for Bigliani type B and C acromions [14].

### Postoperative protocol

Mayo clinic splint was worn for a month, and the rehabilitation protocol began at Day 1 by mobilizing the elbow and the wrist. After the first week and until Week 4, passive movements of the shoulder were allowed up to 120° of extension, 40° external rotation, 90° of abduction and pendular movements. Active movements began at Week 4, and against resistance at Week 12. The self‐rehabilitation protocol described by Liotard was explained to the patient and applied as of Week 4.

### Postoperative analgesics

The same analgesic strategy was used on regular basis postoperatively: ketoprofen 100 mg BID, omeprazole 20 mg once daily, tramadol 50 mg BID, acupan 20 mL QID and paracetamol 1 g QID for the first week. This analgesic strategy contains 10 morphine milligram equivalent per day. In addition to conventional analgesics, a catheter for continuous interscalene infusion of 0.1% Naropin at at 5 mL/h (18 morphine milligram equivalent per day) was placed near the brachial plexus through a subcutaneous tunnel for 3 days. A dedicated provider specifically responsible for ensuring the proper functioning of the Naropin catheter visited the patients daily during the first 3 days. During the postoperative follow‐up, the investigator asked patients over the phone if the catheter functioned as intended. Postdischarge analgesia was prescribed on a round‐the‐clock basis. Compliance with the prescribed regimen after discharge was not reported.

### Outcome measures

The primary end point was the average daily pain level evaluated on a Visual Analog Scale (VAS) from D0 to D5. The VAS was initially completed before surgery while the patient was hospitalized, by a medical student or a resident. After discharge, the patient was called every day by a resident until Day 5 up to complete the VAS over the phone. Surgery was performed on an outpatient basis, except in three cases.

**Figure 1:**
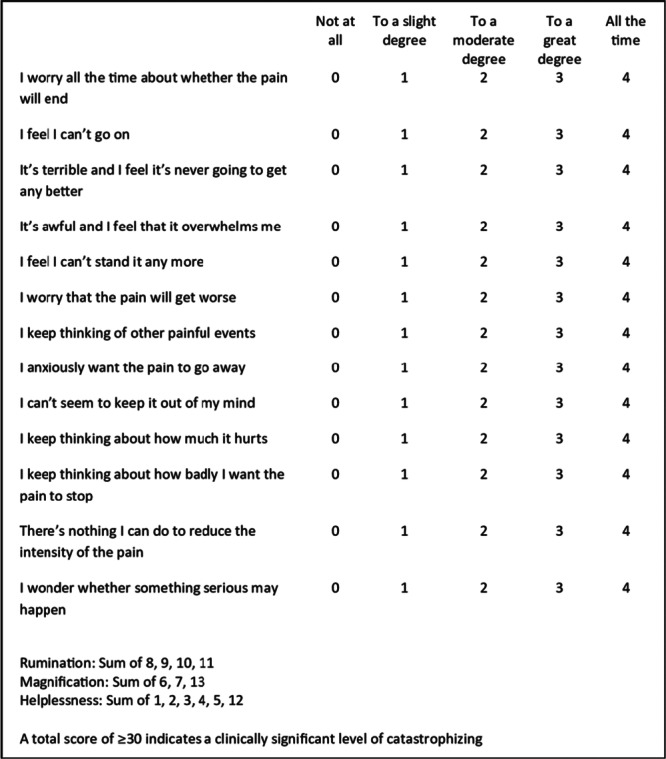
Pain Catastrophizing Scale.

The PCS is a 13‐item scale ranging from 0 to 52, with higher scores indicating greater levels of catastrophizing, as well as increased negative experiences with pain and emotional distress (Figure 1). Answering the questionnaire takes an average of 5 min and was required to be completed on the same day as surgery, before the procedure itself [4, 15, 26, 27].

### Participation and sample size

Sports were defined as playing on regular basis (at least once a week) a sports activity. Manual work was defined as a job requiring physical labour or tasks that require dexterity.

An a priori power analysis was performed to determine whether the available sample size (*n* = 33) was sufficient to detect a statistically significant association between PCS scores and average postoperative pain, as measured by the Visual Analog Scale (VAS) from postoperative Day 0 to Day 5. The analysis was based on a simple linear regression model with one continuous predictor (PCS).

### Statistical analysis

For continuous variables, the mean and standard deviation were reported if they followed a normal distribution, as confirmed by the Shapiro–Wilk test. For continuous variables that did not demonstrate normality, they were described using the median and interquartile range (IQR). Two distinct groups were created based on PCS scores, divided by the median value of the scores in the study population, rather than an established clinical cut‐off. Group 1 included patients with a PCS score less than or equal to the median, representing a low PCS score, while Group 2 included those with a PCS score greater than the median, representing a high PCS score. This division was made to categorize patients based on the distribution of their PCS scores within the sample, rather than using a fixed threshold.

We opted to divide PCS scores using the median value in our sample to better reflect the actual distribution of scores within our relatively small cohort. Given the limited sample size (*n* = 33) and the lack of extreme values approaching the 30‐point threshold, using the median allowed for a more balanced grouping and preserved statistical power for comparisons.

A priori, a power analysis was performed during the study design phase to assess whether the planned sample size would be sufficient to detect a statistically significant association between PCS scores and average postoperative pain (VAS D0–D5). Based on a simple linear regression model with one continuous predictor (PCS), the parameters included an alpha level (*α*) of 0.05, statistical power (1−*β*) of 0.80, and a large effect size (*f*² = 0.35), in accordance with Cohen's guidelines. This analysis indicated a minimum required sample size of 23 participants. With an actual sample of 33 patients, the study was adequately powered (approximately 90%) to detect large effect sizes.

Comparisons between the two groups were conducted using either the Student's *t*‐test or the Wilcoxon test for continuous variables, and the Chi‐square test or Fisher's exact test for categorical variables, depending on the sample size.

A *p*‐value was considered significant if it was less than 0.05. Statistical analyses were performed using R software (version 4.2; R Foundation for Statistical Computing).

### Approval data

Informed consent for participation in this study was obtained from all patients, and the study was approved by the ethics committee IRB‐SOFCOT (Reference 21–2024).

## RESULTS

During the study timeframe of the study, between November 2023 and May 2024 33 patients were operated under arthroscopy for rotator cuff repairs. No patients were excluded; however, one patient never completed the PCS and VAS questionnaire. Demographic data were comparable between the two groups (Table 1).

**Table 1:** Patient demographics.

	Group 0: low PCS, *N* = 16	Groupe 1: high PCS, *N* = 16	*p* value
PCS, mean [SD]	4.6 [4.3]	25.0 [14.4]	<0.001
Age, mean [SD]	58.8 [9.1]	57.0 [8.8]	N.S.
Sex, *N* (%)			N.S.
Female	8 (50%)	9 (56.3%)	
Male	8 (50%)	7 (43.8%)	
Active smokers, *N* (%)	0 (0.0%)	1 (6.3%)	N.S.
Diabetes, *N* (%)	2 (12.5%)	0 (0.0%)	N.S.
Dominant side, *N* (%)	10 (62.5%)	13 (81.3%)	N.S.
Sports, *N* (%)	4 (25.0%)	2 (12.5%)	N.S.
Manual work, *N* (%)	6 (37.5%)	9 (56.3%)	N.S.
Traumatic, *N* (%)	4 (25.0%)	5 (31.3%)	N.S.
Work injury, *N* (%)	2 (12.5%)	1 (6.3%)	N.S.
SSP (stage Patte), *N* (%)			N.S.
1	10 (62.5%)	4 (25.0%)	
2	6 (37.5%)	12 (75.0%)	
Cofiel tear size, *N* (%)			
<1 cm	10 (62.5%)	4 (25.0%)	
1–3 cm	6 (37.5%)	12 (75.0%)	
>5 cm	0 (0%)	0 (0%)	
SSC tear, *N* (%)			N.S.
No tear	12 (75.0%)	11 (68.8%)	
Lafosse 1	6 (37.5%)	5 (31.3%)	
Lafosse 2	3 (18.8%)	2 (12.5%)	
Lafosse 3	0 (0%)	1 (6.3%)	
Repaired	4 (25.0%)	5 (31.3%)	
ISP tear, *N* (%)	4 (25.0%)	4 (25.0%)	N.S.
Biceps procedure, *N* (%)			N.S.
Tenodesis	10 (62.5%)	10 (62.5%)	
Tenotomy	3 (18.8%)	4 (25.0%)	
Acromioplasty, *N* (%)	12 (75.0%)	13 (81.3%)	N.S.
Speed Bridge, *N* (%)	3 (18.8%)	2 (12.5%)	N.S.

Abbreviations: ISP, infraspinatus; N, number of patients; N.S., nonsignificant; PCS, Pain Catastrophizing Scale; SSC, subscapularis; SSP, supra spinatus.

A significant difference in VAS scores was observed between the two groups starting from D2. Three patients had their Naropin catheter dislodged before completing the planned 72 h of analgesia, two were in the low PCS group and one in the high PCS group.

**Figure 2:**
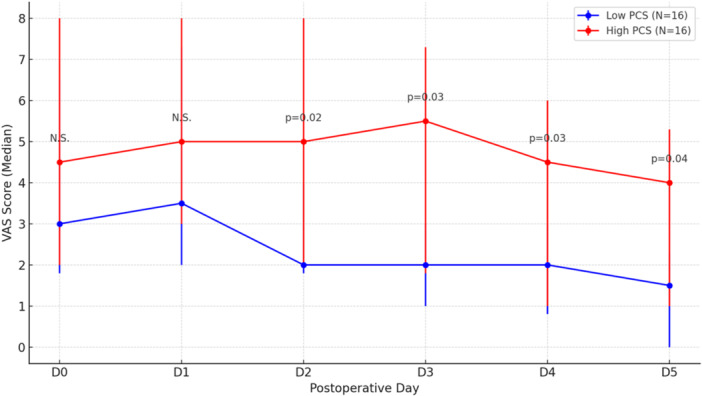
VAS over time by PCS groups.

Patients with a lower preoperative PCS score experienced significantly less pain from the second day onward compared to those with a higher PCS score (Table 2 and Figure 2). The Patient Acceptable Symptom State (PASS) for pain, commonly defined as a VAS score of 3.0 or less, was reached by the low PCS group on postoperative Day 2 (D2), with a median pain score of 2.0 [19, 28, 29]. This group maintained VAS scores below the PASS threshold for the remainder of the study period. In contrast, the high PCS group did not reach the PASS threshold, with their median pain scores remaining above 3.0 throughout the study (5.0 on D2, 5.5 on D3, 4.5 on D4 and 4.0 on D5). These findings suggest that preoperative pain catastrophizing has a significant influence on postoperative pain levels and the time to reach an acceptable symptom state.

**Table 2:** Comparison of postoperative VAS scores between the two groups.

	Group 0: low PCS, *N* = 16	Groupe 1: high PCS, *N* = 16	*p* value
VAS score, median [IQR]			
D0	3.0 [1.8; 6.5]	4.5 [2.0; 8.0]	N.S.
D1	3.5 [2.0; 7.3]	5.0 [3.0; 8.0]	N.S.
D2	2.0 [1.8; 4.0]	5.0 [2.0; 8.0]	0.02
D3	2.0 [1.0; 3.0]	5.5 [1.8; 7.3]	0.03
D4	2.0 [0.8; 3.0]	4.5 [1.0; 6.0]	0.03
D5	1.5 [0.0; 2.3]	4.0 [1.0; 5.3]	0.04

Abbreviations: D, day; IQR, interquartile range; N.S, non significant; PCS, Pain Catastrophizing Scale; VAS, Visual Analog Scale.

## DISCUSSION

Patients with higher preoperative PCS scores reported significantly higher VAS pain scores from the second to the fifth day following surgery. Interscalene catheters remain effective for 3 days, therefore the postoperative pain is between D3 and D5 after the surgery. The higher pain levels observed from D2 onward in the group with greater catastrophizing suggest that pain perception in this group may not be directly related to analgesic efficacy but rather influenced by psychological factors, such as fear and pain apprehension.

Pain catastrophizing seems to influence pain perception and overall recovery, leading to worse postoperative outcomes across various fields. In the context of rotator cuff pathology, Gibson et al. demonstrated that patients with higher PCS scores tend to interpret their condition as more severe [8]. Their study of 107 patients with rotator cuff injuries found a correlation between the PCS and the Western Ontario Rotator Cuff Index (WORC), suggesting that catastrophizing may amplify perceived disability. Similarly, Ponce et al. observed an association between high PCS scores and an increased prevalence of acromioclavicular pain in a study involving 132 patients [20]. Their findings further highlighted the psychological component of pain, identifying a relative risk (RR) of acromioclavicular pain of 1.96 (1.14–3.35) for patients presenting a history of chronic pain, 4.53 (2.05–10.00) for depression, 3.67 (1.66–8.1) for anxiety, and 2.36 (1.29–4.33) for any psychiatric condition. Mental health conditions were not specifically assessed in our study; as these factors may have acted as confounders. The ability to predict postoperative pain control based on preoperative catastrophizing is a useful tool for surgeons. This insight could allow surgeons to adjust their consultations to provide additional reassurance and prepare patients for the early postoperative pain they might experience [25]. Psychological support could also be implemented to reduce anxiety‐provoking thoughts, enabling patients to navigate the early postoperative period under more favourable conditions [10]. For patients with high PCS scores, several interventions could potentially improve outcomes. Preoperative psychological support, such as cognitive behavioral therapy (CBT) or counselling, may help patients reframe catastrophic thoughts and develop effective coping strategies. Combining psychological education with prehabilitation programs, which include physical therapy, could enhance both psychological resilience and physical recovery. Additionally, providing detailed patient education about expected pain levels and recovery processes could reduce anxiety and fear. Pharmacological interventions, such as selective serotonin reuptake inhibitors (SSRIs) or anxiolytics, may also play a role in modulating pain perception. Tailoring pain management strategies to the psychological profiles of patients, for example, through the increased use of regional anesthesia or ketamine, could optimize pain control in high catastrophizers.

For patients with high preoperative PCS scores, adapting pain management strategies may improve recovery. Given the psychological aspect of pain, treatments like SSRIs or SNRIs can reduce sensitivity and improve mood, helping counteract catastrophizing [24]. Preoperative cognitive‐behavioral therapy (CBT) may also foster better coping and reduce anxiety [13], while support groups can build emotional resilience [5]. Combining continuous suprascapular nerve blocks with regional anesthesia may enhance pain control in psychologically sensitive patients [23]. Personalizing care for these individuals addresses both physical and mental aspects of recovery. Future studies should focus on the effectiveness of psychological interventions, such as CBT and mindfulness‐based stress reduction, in reducing postoperative pain. Investigating the impact of PCS scores on long‐term functional outcomes and rehabilitation adherence is also crucial. Furthermore, understanding the complex relationship between catastrophizing, depression, anxiety, and pain perception, and conducting detailed subgroup analyses to identify confounding factors, will be valuable. Finally, research on personalized pain management protocols based on PCS scores could lead to improved patient satisfaction and recovery.

The limitations of this study include the small sample size and the short duration of follow up. Another limitation was the variability in how patients rated their pain on the VAS scale. This variability is influenced by the temporal nature of pain; many patients reported intermittent bursts of intense pain lasting short durations, while their pain was otherwise tolerable throughout the day. Some patients assigned very high scores due to this intermittent discomfort, whereas others gave lower scores due to predominant analgesia. The reliance solely on the VAS as the outcome measure, as well as the lack of reporting on post‐discharge analgesic consumption and the absence of reference to support the use of the cut‐off value we used for PCS are other limitations. The fact that 10 out of 16 patients in the low PCS group and 4 out of 16 patients in the high PCS group suffered from Patte I disease is a limitation.

## CONCLUSIONS

Patients with higher preoperative PCS scores experienced greater early postoperative pain starting from D2.

## AUTHOR CONTRIBUTIONS

Alexandra Stein collected, analysed the data and wrote the manuscript. Alexandre Hardy initiated the study, analysed the data and reviewed the manuscript. Mohamed Moussa reviewed the manuscrupt. Thomas Bauer reviewed the manuscript. Jean‐David Werthel reviewed the manuscript.

## CONFLICT OF INTEREST STATEMENT

The authors declare no conflicts of interest.

## ETHICS STATEMENT

The study was approved by the ethics committee IRB‐SOFCOT (Reference 21–2024). Informed consent for participation in this study was obtained from all patients.

## Data Availability

Data are available upon reasonable request.
